# The clinical and molecular diagnosis of childhood and adolescent pulmonary tuberculosis in referral centers

**DOI:** 10.1590/0037-8682-0205-2020

**Published:** 2020-09-25

**Authors:** Rafaela Baroni Aurilio, Ronir Raggio Luiz, Marcelo Gerardin Poirot Land, Claudete Aparecida Araújo Cardoso, Afrânio Lineu Kritski, Clemax Couto Sant’Anna

**Affiliations:** 1 Universidade Federal do Rio de Janeiro, Departamento de Pediatria da Faculdade de Medicina, Instituto de Puericultura e Pediatria Martagão Gesteira, Rio de Janeiro, RJ, Brasil.; 2 Universidade Federal do Rio de Janeiro, Instituto de Estudos de Saúde Coletiva, Rio de Janeiro, RJ, Brasil.; 3 Universidade Federal Fluminense, Faculdade de Medicina, Departamento de Saúde Materno Infantil, Niterói, RJ, Brasil.; 4 Universidade Federal do Rio de Janeiro, Programa Acadêmico de Tuberculose da Faculdade de Medicina, Instituto de Doenças do Tórax/Hospital Universitário Clementino Fraga Filho, Rio de Janeiro, RJ, Brasil.

**Keywords:** Tuberculosis, Diagnosis, Polymerase chain reaction

## Abstract

**INTRODUCTION:**

The diagnostic accuracy of Xpert MTB/RIF (Xpert) in pulmonary tuberculosis (PTB) in children is lower than in adults. In Brazil, the diagnosis of PTB is based on a diagnostic score system (DSS). This study aims to study the role of Xpert in children and adolescents with PTB symptoms.

**METHODS: A:**

cross-sectional study was conducted in 3 referral centers to TB. Children and adolescents (0-19 years old) whose respiratory samples were submitted to Xpert were included. Statistical analysis (bivariate and logistic regression) to assess the simultaneous influence of TB-related variables on the occurrence of Xpert detectable in TB cases was done. To evaluate the agreement or disagreement between Xpert results with acid-fast bacillus (AFB) and cultures, κ method was used (significancy level of 5%).

**RESULTS:**

Eighty-eight patients were included in the study and PTB occurred in 43 patients (49%) and Xpert was detectable in 21 patients (24%). Adolescents and positive culture results were independent predictive variables of Xpert positivity. DSS sensitivity compared with the final diagnosis of TB was 100% (95% CI, 88.1-100%), specificity was 97.2% (95% CI, 85.5-99.9%). The accuracy of the method was 98.5% (95% CI, 91.7-99.9%).

**CONCLUSIONS:**

Xpert contributed to diagnosis in 9% of patients with AFB and in culture negative cases. DSS indicated relevance for this diagnostic approach of intrathoracic TB (ITB) in reference centers for presenting data both with high sensitivity and specificity.

## INTRODUCTION

In 2018, 72,788 new cases of tuberculosis (TB) were registered in Brazil^1^. According to World Health Organization, out of the 22 countries that represent 80% of TB cases in the world, Brazil occupies 16^th^ position^2^. In 2018, in the state of Rio de Janeiro, the incidence of TB was 66.3 cases/100,000 inhabitants. The percentage of patients that are younger than 14 years was 8% of this total^1^.

Most children who develop primary TB, are paucibacillary and are usually unable to expectorate, hence not allowing bacteriological identification. Since 2002, in Brazil, the diagnosis of intrathoracic TB (ITB) in children is recommended by the National Tuberculosis Control Program (NTCP) based on a diagnostic scoring system (DSS) that includes clinical symptoms, epidemiological TB history, tuberculin skin test (TST) result, radiological findings, nutritional state, and does not require bacteriological confirmation^3^ ^,^ ^4^. Based on the final punctuation (≥ 40 points (very likely pulmonary TB); 30-35 points (possible pulmonary TB); ≤ 25 points (unlikely pulmonary TB)), it allows for the start of a treatment for ITB according to the total points. After the implementation of this strategy, several studies carried out its validation and obtained sensitivity greater than 80% and specificity between 70-90%^5^. More details about DSS are shown in Supplementary Figure 1.

Since 2014, the molecular diagnosis of TB in Brazil is conducted through Xpert MTB/RIF system (Xpert)^6^. In the meta-analysis study conducted by Detjen et al.^7^ comparing 15 ITB studies conducted in children and adolescents, the sensitivity and specificity of Xpert compared to the cultures, were 62% (95% confidence interval [CI], 51-73%) and 98% (95% CI, 97-99%) in spontaneous or induced sputum (IS), respectively, and for gastric lavage (GL), 62% (95% CI, 51-73%) and 98% (95% CI, 96-99%), respectively. Compared with acid-fast bacilli (AFB) smear test, Xpert sensitivity was 36% higher in sputum/induced sputum (IS) samples and 44% higher in GL samples. The detection limits of Xpert in children is lower than in adults with ITB. In another systematic review^8^, the positivity of TB based on Xpert among children ranged from 2-17% in IS, 5-51% in GL, and 3-8% in nasopharyngeal aspirate (NPA).

The aim of this study was to describe the diagnostic assessment of ITB and the role of Xpert in children and adolescents with ITB symptoms who visited referral centers in the state of Rio de Janeiro, Brazil.

## METHODS

The study was cross-sectional. The data were collected in three referral centers of TB situated in the state of Rio de Janeiro (Hospital Municipal Raphael de Paula e Souza, Hospital Universitário Antônio Pedro of Fluminense Federal University and Instituto de Puericultura e Pediatria Martagão Gesteira at Federal University of Rio de Janeiro) from October 2014 to March 2019. 

Patients aged 0-19 years with clinical-radiological symptoms of ITB were included in the study and one respiratory sample from these patients that could reflect ITB disease was submitted to Xpert^9^. The specimen could be sputum, IS, GL, bronchial lavage, bronchoalveolar lavage (BAL), and pleural effusions (PE). Patients whose clinical specimens were insufficient or contaminated for analysis were excluded.

Patients with ITB symptoms were evaluated by physicians from hospitals and had sub-acute or chronic respiratory infections (≥ 14 days) and abnormal chest radiographs. They may or may not have other elements suggestive of ITB, such as contact with adults with ITB, positive TST, and undernutrition. Only 1 sample from each patient underwent Xpert test.

For each patient included in the study, data were collected and DSS^4^was performed retrospectively by the main researcher (RBA). The variables analyzed were age, history of contact with ITB (in the last 2 years), and DSS result (very probable TB when the sum was ≥ 40; possible ITB, 30 or 35; ITB unlikely, ≤ 25 points)^4^, TST (negative and positive), Xpert result (detectable and undetectable), smear AFB, and mycobacterial culture in respiratory specimens.

Through clinical, radiological, and laboratory criteria during the first care and follow-up at referral centers, the patients were classified after 60 days of follow-up according to the treatment responses as either final diagnosis of TB - favorable evolution with anti-TB treatment, or non-TB - with other pulmonary diseases that show satisfactory evolution without anti-TB treatment.

Although the Ministry of Health in Brazil^4^ ^,^ ^10^adopts an age of 10 years to categorize children and adolescents, in this article, we consider the following: children are patients younger than 11 years and adolescents are those aged 11 to 19 years (cutoff point selected by ROC curve). 

A database was elaborated using the Microsoft Office Excel program (2010 version) and data analysis was performed using the SPSS statistical software package v 21. Data analysis was performed using descriptive statistics, with frequencies for continuous variables and percentages for categorical variables. For bivariate analysis, statistical significance was calculated using the χ^2^ test or Fisher’s test whenever appropriate. The level of significance was set to 5%. Logistic regression analysis was performed to assess the simultaneous influence of TB-related variables on the occurrence of detectable Xpert values in cases which indicate a final diagnosis of TB. The choice of variables for modeling was based on the significance level of bivariate analysis of up to 20%. The collinearity between the candidate variables which enter the model was evaluated by a correlation matrix. The cutoff point, which was used to dichotomize the continuous variable age, was determined by the receiver operating characteristic (ROC) curve. Sensitivity and specificity of DSS relative to final ITB diagnosis were performed using scores ≥ 30 or < 30 points. To evaluate the agreement between Xpert results (detectable or undetectable) with AFB smear test and culture (results from both the methods were classified as positive or negative), κ method was used.This project was approved by the Research Ethics Committee of IPPMG/Universidad e Federal do Rio de Janeiro (961.452).

## RESULTS

Eighty-eight patients with ITB symptoms were included in the study. The median age of patients was 105 months (interquartile range [IQR]: 34-168 months). Contact with TB was reported in 41 (53%) of 77 patients and positive TST was observed in 25 (39%) of 64 patients. There were no cases of ITB associated with extra-thoracic TB . Of the 88 patients studied, 48 (54%) were children, and 40 (46%) were classified as adolescents.

**FIGURE 1:**
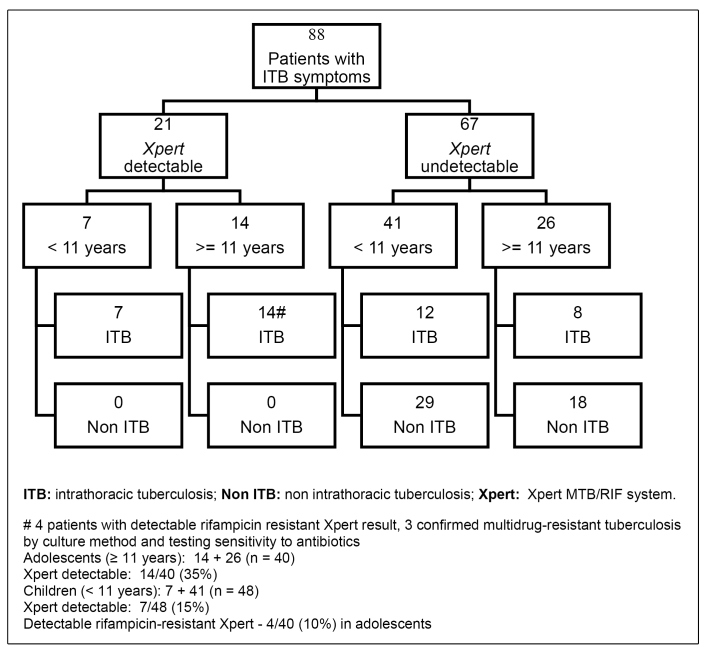
Population distribution in relation to Xpert test results in 88 patients with intrathoracic tuberculosis symptoms.

Of the 88 patients included, 65 had DSS analyzed by the main researcher (RBA): 23 patients (35%) achieved 40 points or more; 7 patients (11%) were between 30 and 35 points; and 35 patients (54%) had 25 points or less. Xpert was detectable among 21 (24%) of 88 cases, positive AFB smears were identified in 10 cases (12%), and culture in 17 (20%) (Table 1). Resistance result by Xpert was observed in 4 (10%) out of 40 adolescents, and 4 (19%) out of 21 total patients with positivity by Xpert. The population distribution in relation to Xpert positivity is shown in Figure 1.

**TABLE 1:** Bivariate analyses showing clinical and laboratory characteristics in children and adolescents with intrathoracic tuberculosis symptoms.

		Patients with ITB symptoms n=88							
		Children (n=48)				Adolescents (n=40)			
		ITB	Non ITB	Total	***p* value**	ITB	Non ITB	Total	***p* value**
		(n=21)	n=27)			(n=22)	(n=18)		
TST (n=64)	Positive	7(46.7%)	4(17.4%)	11	0.073*	10 (83.3%)	4(28.6%)	14	0.005
	Negative	8(53.3%)	19(82.6%)	27		2(16.7%)	10(71.4%)	12	
Contact with PTB (n= 77)	Yes	14(66.7%)	9(37.5%)	23	0.051	11(68.8%)	7(43.8%)	18	0.154
	No	7(33.3%)	15(62.5%)	22		5(31.2%)	9(56.2%)	15	
Xpert (n=88)	Detectable	7(33.3%)	0	7	0.002*	14 (63.6%)	0	14	<0.001
	Undetectable	14(66.7%)	27(100%)	41		8 (36.4%)	18(100%)	26	
AFB (n=82)	Positive	3(15.0%)	1(4.0%)	4	0.309*	6(30.0%)	0	6	0.022*
	Negative	17(85.0%)	24(96.0%)	41		14(70.0%)	17(100%)	31	
Culture n=83)	Positive	8(42.1%)	0	8	<0.001*	9(40.9%)	0	9	0.005*
	Negative	11(57.9%)	26(100%)	37		13(59.1%)	16(100%)	29	
DSS (n=65)	≥30	18(100%)	0	18	<0.001	11 (100%)	1(7.7%)	12	<0.001
	<30	0	23(100%)	23		0	12(92.3%)	12	

**ITB:** intra-thoracic tuberculosis; **Non ITB:** non- intrathoracic tuberculosis; **TST:** tuberculin skin test; **PTB:** pulmonary tuberculosis; **Xpert:** Xpert MTB-RIF system; **AFB:** acid-fast bacilli; **DSS:** diagnostic scoring system.

 Xpert was detectable in 7 (15%) out of 48 children, including 3/7 samples from BAL, 3/7 specimens from GL, and 1/7 from PE samples, and 14 (35%) out of 40 adolescents, with 11/14 from specimens taken from the sputum and IS and the other 3 samples were from PE and BAL. The final diagnosis of ITB was established in 43 (49%) of the 88 cases. The characteristics of ITB and non-ITB patients are described in Table 1.

In 25 (58%) of the 43 patients with ITB, the time from specimen collection to the delivery of the Xpert results to the health team ranged from 0 to 22 days, with a median of 1 day (IQR: 1-6 days). In 10 (40%) of these 25 cases studied, treatment was started before receiving the laboratory Xpert results.

The comparison among Xpert, AFB smear and culture techniques are described in Table 2. In 81 patients who did all three methods simultaneously, Xpert was detectable in 7 patients who were ABF and culture negative. The Supplementary Table 1 shows the results of Xpert compared with TB-related characteristics in the group with final TB diagnosis (bivariate analysis). Based on these results, logistic regression analysis was performed.

**TABLE 2:** Comparison of Xpert results with acid-fast bacilli and culture methods

Bacteriological results		Patients with ITB symptoms			
		*Xpert*	*Xpert*	Total	*kappa*
		Detectable (n= 21)	Undetectable (n= 67)		(CI 95%)
AFB (n=82)	Positive	5(26.3%)	5(8.0%)	10	0.220
	Negative	14(73.7%)	58(92.0%)	72	(0.194 - 0.247)
Culture (n=83)	Positive	13(61.9%)	4(6.5%)	17	0.592
	Negative	8(38.1%)	58(93.5%)	66	(0.537 - 0.646)

**ITB:** intrathoracic tuberculosis; **AFB:** acid-fast bacilli; **Xpert:** Xpert MTB/RIF system.

In the multivariate analysis, it was observed that patient age older than 11 years and positive culture result were independent characteristics of Xpert positivity (Table 3). In 35 patients with DSS score of less than 30, Xpert was undetectable in 100% of cases. In 30 patients with a score of 30 or greater, Xpert was detectable in 15 cases (50%). DSS sensitivity compared with the final diagnosis of TB was 100% (95% CI, 88.1-100%), specificity was 97.2% (95% CI, 85.5-99.9%) and the accuracy of this method was 98.5% (95% CI, 91.7-99.9%). 

**TABLE 3:** Multivariate analyses: the association of variables with a higher chance for Xpert positivity.

Predictor characteritic	OR	95% CI	***p* value**
Positive culture	8.820	1.751 - 39.186	0.008
Age ≥ 11 years	4.177	0.926 - 18.846	0.063
Constant	0.211		0.024

**OR:** Odds ratio; **CI:** confidence interval.

## DISCUSSION

Our study was conducted in three referral centers for children and adolescents with ITB in the state of Rio de Janeiro. The positive AFB smear result of 12% obtained in our study is higher than the values obtained by previous studies which ranged ​​between 1.6-4.8%^11^ ^,^ ^12^ ^,^ ^13^. Comparing the three methods used in our study, almost 25% of our patients with detectable Xpert also had a positive AFB smear with little agreement by κ index. This finding is similar to a previous study that showed higher performance of a molecular test than AFB smear test for the diagnosis of ITB among children and adolescents^13^. On the other hand, more than half of the patients with detectable Xpert also had a positive culture result, which could be explainable since the detection limit of the culture method could range from 10 to 100 colony-forming units (CFUs)/mL and approaches the values of the molecular test, which is 131 CFUs/mL^14^. Xpert contributed to diagnosis in 7 cases (9%) where both AFB and culture methods were negative. The evaluation of Xpert results exclusively in pulmonary specimens from patients who were younger than 15 years in South Africa^15^, showed similar positivity to our culture result (71.4% of the samples with detectable Xpert in IS and 65.1% in NPA). The evaluation of symptomatic contacts with active ITB disease cases in West Africa^11^ detected lower Xpert positivity result of 42% in samples with positive culture. 

In the present study, Xpert positivity was 24%, which is much higher than the 2.4% value obtained by Togun et al. in West Africa^11^ analyzing IS samples from patients younger than 15 years who had previous contact with active ITB patients or respiratory symptoms and/or positive TST. It is possible that the low positivity of this study compared with our study was due to the fact that only IS was used which could generate samples with low bacillary load in children. Similarly, based on studies in India, a low positivity rate of Xpert was observed. Raizada et al.^12^ obtained 10.4% Xpert positivity in referral centers among symptomatic respiratory patients without compatible radiological imaging, and Das et al.^13^, analyzing samples from GL, IS, cerebrospinal fluid and lymph node from patients who are younger than 15 years, found only 11% Xpert positivity. In these studies, Xpert positivity lower than what was observed by our study could be due to the fact that, in the first study, the authors included only patients under 15 years of age with TB symptoms without enhancing the radiological and, in the second study, the authors indistinctly analyzed respiratory and extrapulmonary specimens. The inclusion criteria adopted by us were stricter than those adopted by other authors including cases with signs and symptoms suggestive of TB associated with other radiological findings suggestive of the disease, which increased Xpert positivity. On the other hand, in the meta-analysis study performed by Detjen et al.^7^ in patients aged 0 to 15 years with presumed PTB, with or without suggestive radiological imaging, Xpert positivity was measured to be 11%.

Specifically analyzing the Xpert positivity of 15% observed among children, the highest detection rates occurred in specimens collected from GL and BAL. Ioos et al.^9^ in a systematic review with children younger than 15 years, observed that Xpert detection in respiratory specimens from patients with presumed ITB was higher in GL samples, however, BAL and PE were not evaluated. 

Among the adolescents included in the present study, Xpert positivity was observed to be 35%, which is higher than 15.7% found in the study conducted in Rio de Janeiro City^16^, which included adolescents with ITB symptoms, in the year of the implementation of the laboratory method as routine procedure in TB bacteriological identification. Lower Xpert performance was expected since any person to be evaluated for ITB was included in the study, regardless of their epidemiological history, laboratory results, and chest radiograph imaging results.

In the current study, 10% of drug resistance was detected among adolescents with ITB symptoms. Analyzing rifampicin resistance (RIF-resistance) detected by Xpert among cases in which this method was positive, our result was 19%, similar to the 16.6% reported by Raizada et al.^13^, in India for patients under 14 years of age. In both these studies, respiratory samples were exclusively analyzed and the studies were conducted in TB units. 

Analyzing the results based on age groups, the entire children group with ITB presented a DSS result of 30 or greater, allowing the start of anti-TB treatment. In contrast, in those individuals with no ITB, all of them had a score of less than 30. In the ITB group, bacteriological identification was observed among 33% of the patients, although this age group typically is paucibacillary. Also, in adolescents with ITB, almost every population had DSS value of 30 or greater, and a little more than half the population had detectable Xpert results. These findings support the validation of DSS obtained in other studies and, at the same time, guide the careful use of Xpert, preceded by DSS, in children and adolescents treated at referral centers^3^ ^,^ ^17^ ^,^ ^18^. DSS has been standardized for over a decade in Brazil. Our data confirmed the high sensitivity and specificity of DSS, and it would be justifiable to routinely adopt this system to make a decision to start ITB treatment. Diagnosis without bacteriological or molecular confirmation is still a reality in endemic areas of TB. A study conducted by Oliveira et al^19^in Rio de Janeiro analyzing adolescents showed that the clinical diagnosis of TB was decisive in patients with greater complexity (diagnosis at hospital level, carriers of human immunodeficiency virus, or combined forms of TB) and they had a negative Xpert value. In addition, the time from clinical sample collection to start of treatment by the health care team varied from 8 days (IQR, 6-14 days) in the Xpert-positive/culture-positive group to 12 days (IQR, 5-36 days) in the Xpert-negative/culture-negative group. In contrast, our shortest interval occurred since this study was conducted at a referral center. In addition, we found that 40% of the anti-TB treatment group was instituted based on the clinical presentation and DSS, indicating its relevance on the diagnostic approach of ITB in this population in primary care units, such as referral centers, for presenting the results with both high sensitivity and specificity^3^.

Although the positive culture and age above 11 being independent variables for Xpert positivity, the small sample size has OR with very wide 95% CI, meaning low precision. 

One of the limitations was the collection of a only a single sample from each patient for Xpert analysis. Perhaps Xpert positivity percentages would be higher if more than one sample was collected per patient^8^. Another limitation of the study is that it was conducted only in referral centers, not including basic health units. It is probable that this situation provided a classification bias since we used more criteria to select eligible patients, generating greater positivity of laboratory methods in the pediatric group. There is a perspective to increase the diagnostic capacity of new molecular tests via Xpert Ultra, which was built recently using the same methodology as Xpert, but with higher sensitivity due to its lower detection limit for *Mycobacterium tuberculosis* (similar to the lower limit of culture)^20^ ^,^ ^21^. Its use in combined specimens might be able to increase the sensitivity in cases of bacteriologically confirmed ITB, but in cases with low bacillary load, the detection of RMP resistance might be impaired^22^.

Future studies with patients from primary care units in our country might provide a comparison of Xpert results with our patients.
