# Supplementary figures and images for: The clinical and molecular diagnosis of childhood and adolescent pulmonary tuberculosis in referral centers
Source: Rev Soc Bras Med Trop. 2020 Sep 25;53:e20200205. doi: 10.1590/0037-8682-0205-2020 (PMC7523522; doi:10.1590/0037-8682-0205-2020)

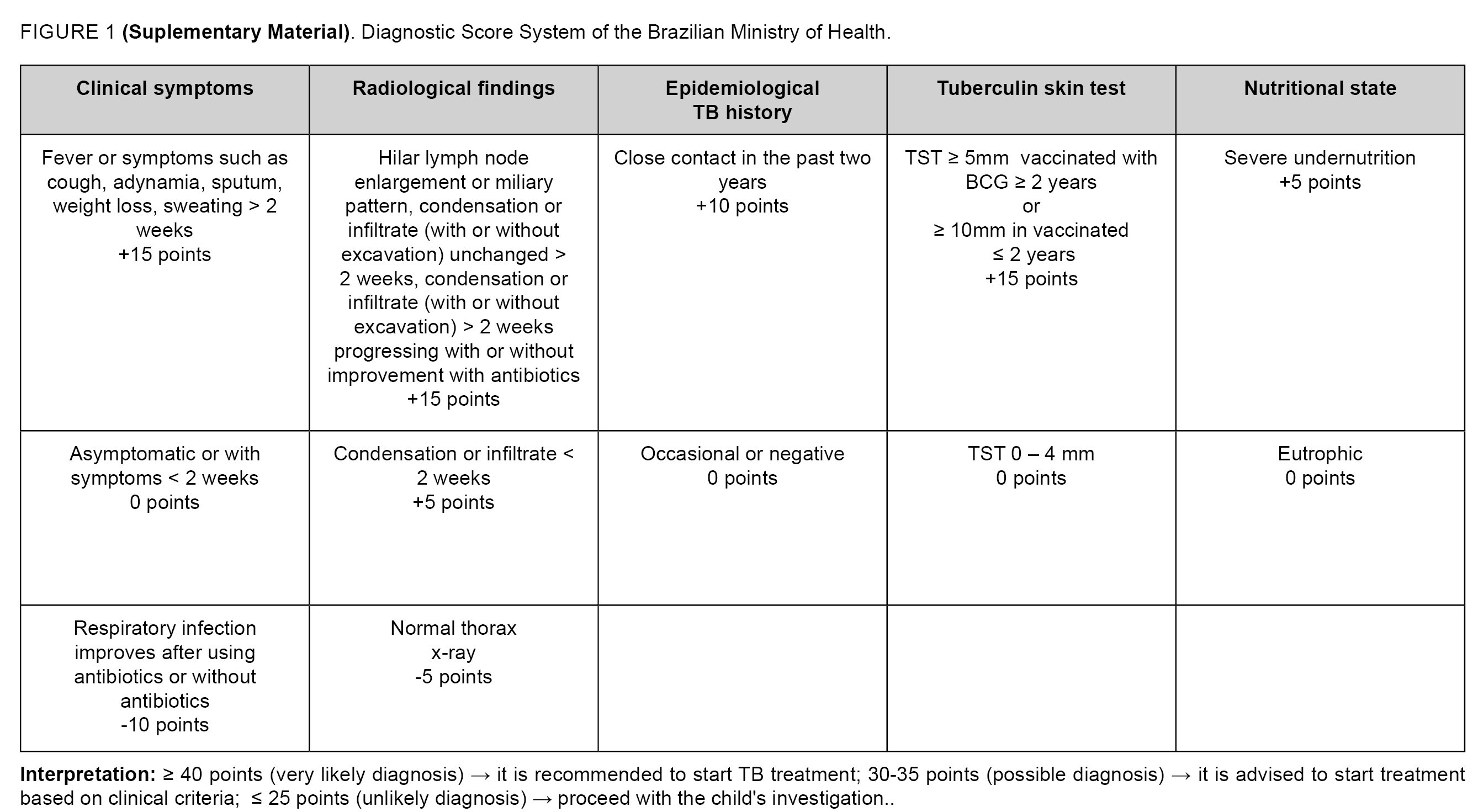

Supplement: Supplementary file 1 [file 1678-9849-rsbmt-53-e20200205-suppl1.jpg]

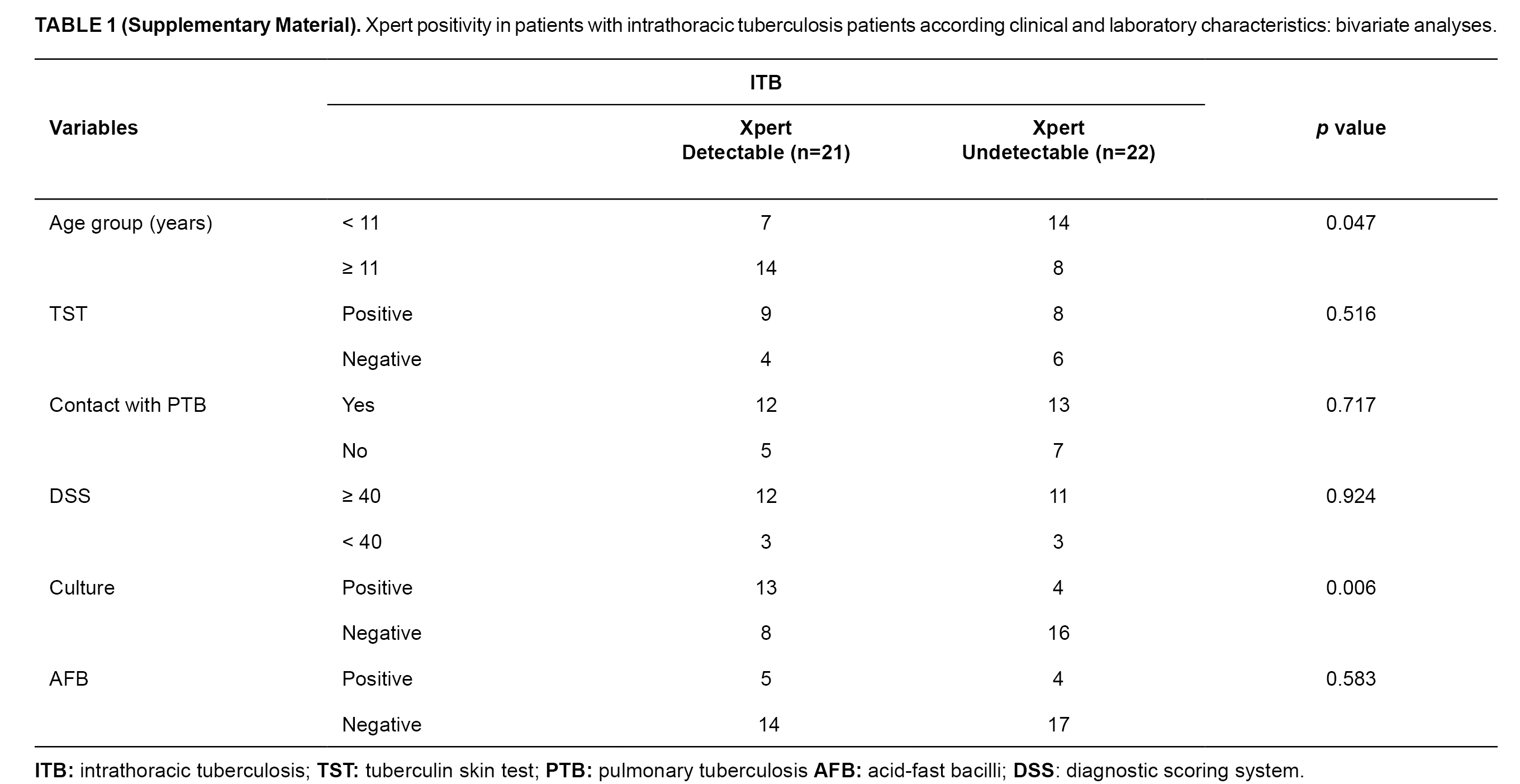

Supplement: Supplementary file 2 [file 1678-9849-rsbmt-53-e20200205-suppl2.jpg]
